# A prolonged multispecies outbreak of IMP-6 carbapenemase-producing Enterobacterales due to horizontal transmission of the IncN plasmid

**DOI:** 10.1038/s41598-020-60659-2

**Published:** 2020-03-05

**Authors:** Takuya Yamagishi, Mari Matsui, Tsuyoshi Sekizuka, Hiroaki Ito, Munehisa Fukusumi, Tomoko Uehira, Miyuki Tsubokura, Yoshihiko Ogawa, Atsushi Miyamoto, Shoji Nakamori, Akio Tawa, Takahisa Yoshimura, Hideki Yoshida, Hidetetsu Hirokawa, Satowa Suzuki, Tamano Matsui, Keigo Shibayama, Makoto Kuroda, Kazunori Oishi

**Affiliations:** 1 Infectious Disease Surveillance Center, National Institute of Infectious Diseases, Tokyo, Japan; 2 Antimicrobial Resistance Research Center, National Institute of Infectious Diseases, Tokyo, Japan; 3 Pathogen Genomics Center, National Institute of Infectious Diseases, Tokyo, Japan; 4 Field Epidemiology Training Program, National Institute of Infectious Diseases, Tokyo, Japan; 5 Department of Infectious Diseases, National Hospital Organization Osaka National Hospital, Osaka, Japan; 6 Infection Control Team, National Hospital Organization Osaka National Hospital, Osaka, Japan; 7 Department of Surgery, National Hospital Organization Osaka National Hospital, Osaka, Japan; 8 Department of Pediatrics, National Hospital Organization Osaka National Hospital, Osaka, Japan; 9 Osaka City Public Health Office, Osaka, Japan; 10 Department of Bacteriology II, National Institute of Infectious Diseases, Tokyo, Japan

**Keywords:** Medical genomics, Medical genomics, Bacterial infection, Bacterial infection, Epidemiology

## Abstract

A multispecies outbreak of IMP-6 carbapenemase-producing Enterobacterales (IMP-6-CPE) occurred at an acute care hospital in Japan. This study was conducted to understand the mechanisms of IMP-6-CPE transmission by pulsed-field gel electrophoresis (PFGE), multilocus sequence typing and whole-genome sequencing (WGS), and identify risk factors for IMP-6-CPE acquisition in patients who underwent abdominal surgery. Between July 2013 and March 2014, 22 hospitalized patients infected or colonized with IMP-6-CPE (*Escherichia coli* [n = 8], *Klebsiella oxytoca* [n = 5], *Enterobacter cloacae* [n = 5], *Klebsiella pneumoniae* [n = 3] and *Klebsiella aerogenes* [n = 1]) were identified. There were diverse PFGE profiles and sequence types (STs) in most of the species except for *K. oxytoca*. All isolates of *K. oxytoca* belonged to ST29 with similar PFGE profiles, suggesting their clonal transmission. Plasmid analysis by WGS revealed that all 22 isolates but one shared a ca. 50-kb IncN plasmid backbone with *bla*_IMP-6_ suggesting interspecies gene transmission, and typing of plasmids explained epidemiological links among cases. A case-control study showed pancreatoduodenectomy, changing drains in fluoroscopy room, continuous peritoneal lavage and enteric fistula were associated with IMP-6-CPE acquisition among the patients. Plasmid analysis of isolates in an outbreak of IMP-6-CPE suggested interspecies gene transmission and helped to clarify hidden epidemiological links between cases.

## Introduction

Carbapenem-resistant Enterobacterales (CRE) is one of the most worrisome antimicrobial-resistant pathogens because of the severity of disease caused, rapid spread across the world, potential to spread into the community, shortage of effective drugs and lack of drugs under development^1–3^. The spread of certain clonal strains and epidemic resistance plasmids that carry the gene coding carbapenemase is considered to be a driving force of its rapid spread^1,4^. However, the distribution of clonal strains of each species and the types of epidemic resistance plasmids and carbapenemase are geographically diverse^4–6^.

CRE is still rare in Japan, where meropenem resistance (minimum inhibitory concentration [MIC] ≥4 μg/mL) was 0.2% among *Escherichia coli* and 0.5% among *Klebsiella pneumoniae* in 2016 based on the National Surveillance^7^. In Japan, the predominant carbapenemase detected in Enterobacterales is an IMP type metallo-β-lactamase (MBL), and sporadic cases or small outbreaks of IMP producers have been reported across the country^8–12^. IMP producers are resistant to almost all β-lactam antibiotics, which limits treatment options. IMP-6 MBL does not hydrolyse imipenem efficiently, and its producer is usually susceptible to imipenem *in vitro* (MIC ≤ 1 μg/mL)^8,9^, which may be a risk that can be ignored in clinical laboratories. Because there is no established evidence that infection due to IMP-6 producer is treatable with imipenem, we consider it is important to control bacteria harbouring *bla*_IMP-6_ to avoid their spread without our awareness.

In July 2010, a patient with CRE was first identified at Osaka National Hospital (ONH). Subsequently, patients infected or colonized with CRE continued to be identified at the hospital despite enhanced control measures taken by the hospital infection control team. Because of the continued detection of CRE, which totalled more than 100 cases between 2010 and 2014, and affected neighbouring healthcare facilities, an investigation was initiated by the Osaka City Public Health Office (OCPHO) and the National Institute of Infectious Diseases (NIID) as a public health response in March 2014. The majority of isolates had *bla*_IMP-6_ and belonged to various species of Enterobacterales.

The objectives of this study were to describe the features of this long-standing outbreak of IMP-6 carbapenemase-producing Enterobacterales (IMP-6-CPE) outbreak through a plasmid analysis and to identify risk factors of its acquisition among patients who underwent abdominal surgery in ONH.

## Materials and Methods

### Setting and case definition

ONH is a tertiary referral hospital with approximately 700 beds in Osaka, Japan. It examines more than 200,000 patients every year, the majority of whom are referred from hospitals in Osaka and neighbouring prefectures. Because this investigation was conducted as a public health response to the outbreak, no informed consent was obtained from the study population. The ethical committee waived the need for written consent for the research handling the bacterial isolates.

Because the epidemiology of the outbreak and risk factors of acquiring IMP-6-CPE might be different between the early stage around 2010 and the latest stage around 2014, we focused on the cases during the nine months prior to the public health response. A case was defined as a hospitalized patient who tested positive for IMP-6-CPE from a clinical specimen at ONH between 1 July 2013 and 15 March 2014. Medical records and surgical records were reviewed for laboratory data, patient demographics characteristics and medical procedures for six months before IMP-6-CPE detection. An epidemiological link was defined as a patient sharing the same ward with another patient for at least one day, and the two wards of the Department of Surgery was considered as the same ward (E9 and W9).

### Bacterial isolates

Clinical isolates of Enterobacterales identified as resistant to carbapenem (imipenem and/or meropenem ≧8 μg/mL) or resistant to both broad-spectrum β-lactams and cephamycins by a Phoenix automated microbiology system (BD Japan, Tokyo, Japan) were screened for MBL production by a double-disk synergy test with sodium mercaptoacetate^13^ at ONH. The double-disk synergy test positive isolates at ONH were defined as suspected MBL producing Enterobacterales, and further subjected to PCR^14^ and Sanger sequencing at NIID to confirm the presence of *bla*_IMP-6_. A conjugation experiment was performed using *E. coli* DH10B as a recipient by the broth-mating method. The conjugants were selected on LB agar plates containing streptomycin (800 μg/mL) and ceftazidime (16 μg/mL).

### Pulsed-field gel electrophoresis (PFGE)

Bacterial genomic DNA was prepared in an agarose block and digested with *Xba*I (New England Biolabs, MA, USA) for *Klebsiella oxytoca*, *K. pneumoniae* and *E. coli* and *Spe*I (New England Biolabs) for *Enterobacter cloacae*. The DNA fragments were separated in a 1% agarose slab gel by a CHEF Mapper system (Bio-Rad, CA, USA) for 24 h with a ramped pulse time of 12.6–40.1 s. DNA bands larger than 48.5 kb were detected automatically using GelCompar II software, version 6.6 (Applied Maths, St-Martens-Latem, Belgium). The Dice coefficient was used to calculate similarities, and UPGMA was used for cluster analysis.

### Whole-genome sequencing (WGS) of plasmids and multilocus sequence typing (MLST)

S1 nuclease-PFGE was performed as described previously^15^. Briefly, an agarose plug containing a bacterial genomic DNA was treated by S1 nuclease to digest circular forms of the plasmids, resulting in linearized forms. All visible plasmid and chromosomal DNA bands were excised from S1-PFGE agarose gel, and the purified DNA was subjected to DNA-Seq for paired-end short reads (2 × 300 mer) with an MiSeq Reagent Kit v3 (Illumina, San Diego, CA, USA), followed by *de novo* assembly by A5-miseq ver. 20140604^16^. The complete circular plasmid sequences were determined by long reads on a Sequel sequencer (PacBio, Menlo Park, CA, USA), followed by *de novo* assembly and error correction by Canu version 1.4^17^, minimap version 0.2-r124^18^, Racon version 1.1.0^19^, Circlator version 1.5.3^20^ and Pilon version 1.18^21^. Gene extraction and annotation were performed by Prodigal version 2.63^22^, and homology searching against a public nucleotide database (NCBI nr), respectively.

To identify the bacterial sequence type, antimicrobial resistance gene and plasmid replicon type, the assembled contigs were analysed by MLST **2.0**^23^ and ResFinder **3.2**^24^, and PlasmidFinder **2.1**^25^, respectively.

The hierarchical cluster analysis of conserved plasmid genes was performed by usearch version 8.1.1812 with the following parameters after sorting by amino acid sequence length: -cluster_smallmem -minsl 0.8 -minqt 0.8 -maxqt 1.25 -query_cov 0.8 -target_cov 0.8^26^, followed by visualization based on the heatmap.2 program in the gplot R package with Pearson correlation and ward.D2 clustering method. Plasmid clustering type based on conserved gene patterns was distinguished at a similarity threshold of 74%. The complete and draft sequences of the plasmids carrying *bla*_IMP-6_ were deposited in DDBJ/EMBL/GenBank as shown in the Supplementary Table.

### Case-control study

Because half of the cases were patients who were admitted to the Department of Surgery, a case-control study was conducted to establish risk factors of IMP-6-CPE acquisition among patients who underwent abdominal surgery at ONH. From the observation of infection control practices in the wards of ONH, we hypothesized that suboptimal infection control practices when dealing with surgical drains and providing wound care might have played a central role in the acquisition of IMP-6-CPE. A case in this case-control study was defined as a hospitalized patient who tested positive for IMP-6-CPE that carried *bla*_IMP-6_ in the abdominal wound or drain discharge after abdominal surgery during the study period. A control was defined as a hospitalized patient who tested positive for meropenem-susceptible Enterobacterales in the abdominal wound or drain discharge after abdominal surgery during the same period and was selected randomly from the surgical records at a 1:2 ratio.

### Statistical analysis

For the case-control study, we used the Mann-Whitney U-test and Fisher’s exact test as appropriate. To test the variables with rare event and for adjustment by days of hospitalization, we used exact logistic regression. A two-tailed P <0.05 was considered to be statistically significant. Stata 14 (Stata Corp., College Station, TX, USA) was used for all statistical analyses.

## Results

### Description of cases

**Figure 1:**
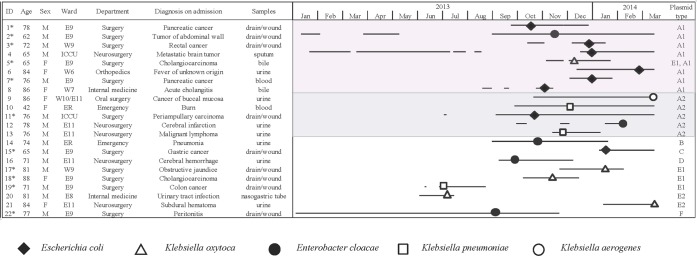
Basic characteristics of cases of IMP-6 carbapenemase-producing Enterobacterales and its isolates, Osaka, Japan, June 2013-March 2014, and the timeline of detection. Horizontal lines indicate durations of hospitalization and nodes indicate detection of isolates. Asterisks indicate the cases included in the case-control study. The cases with type A1 plasmid are shown on a background of pink, and the cases with type A2 plasmid are shown on a background of blue. Case 9 was admitted to ward E11 of the Department of Neurosurgery on 20 January and was treated there until 17 February 2014. ICCU: intensive cardiac care unit.

Among the 29 inpatients with suspected MBL producing Enterobacterales identified at ONH during the study period, 22 IMP-6-CPE cases were confirmed (Fig. 1). The median age of these 22 patients was 76 years old (interquartile range [IQR]: 71–81) and 15 (68%) were men. The diagnosis on admission was malignant or benign solid tumours (50%), infectious diseases other than CRE infection (18%) or cerebrovascular disorders (14%), and all but one case (case 15 with isolates from the nasogastric tube) were considered to have developed infection due to IMP-6-CPE. Half of the cases (50%) were hospitalized in the Department of Surgery, followed by Neurosurgery (23%), Emergency Medicine (9%) and Internal Medicine (9%). IMP-6-CPE was isolated most frequently from abdominal surgical wounds or drains (41%) followed by urine (32%). The isolates consisted of five different species of Enterobacteriaceae including *E. coli* (n = 8, 36%)*, K. oxytoca* (n = 5, 23%)*, E. cloacae* (n = 5, 23%)*, K. pneumoniae* (n = 3, 14%) and *K. aerogenes* (n = 1, 5%).

### PFGE and MLST

**Figure 2:**
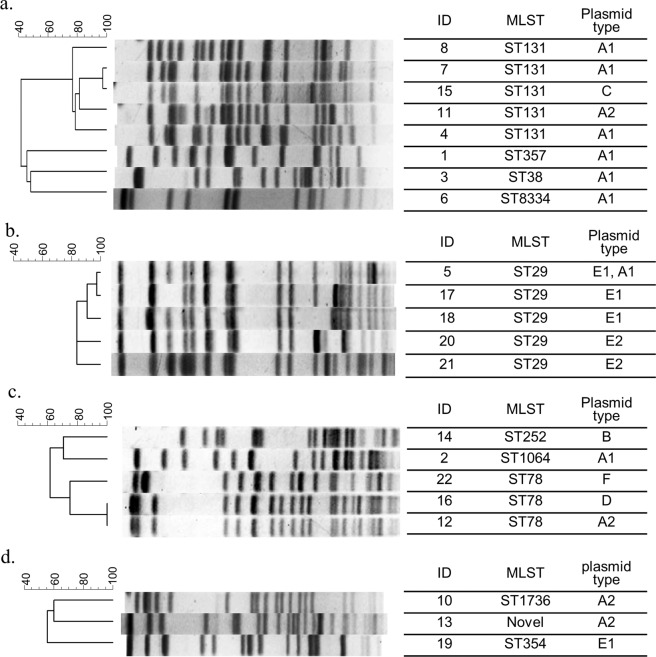
Dendrogram of pulsed-field gel electrophoresis (PFGE) profiles, multilocus sequence typing (MLST) data and plasmid types for the 22 isolates from IMP-6 carbapenemase-producing Enterobacterales cases. (**a**) *Escherichia coli*, (**b**) *Klebsiella oxytoca*, (**c**) *Enterobacter cloacae*, (**d**) *Klebsiella pneumoniae*. MLST:Multilocus sequence typing.

The results of PFGE and MLST are shown in Fig. 2. Five of the eight isolates of *E. coli* were ST131, the global epidemic clone^5^, and other isolates showed diverse PFGE profiles and sequence types (STs) (Fig. 2a). All five isolates of *K. oxytoca* were considered to be clonally related and were ST29 (Fig. 2b). Three isolates of *E. cloacae* were ST78, and others had distinctly different PFGE profiles and STs (Fig. 2c). The PFGE profiles and STs of *K. pneumoniae* were diverse (Fig. 2d).

### Analysis of plasmid

**Figure 3:**
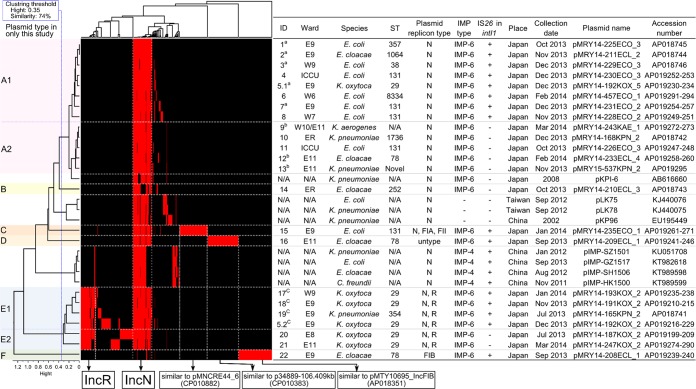
Possible relationship between 23 plasmid types in the 22 isolates from IMP-6 carbapenemase-producing Enterobacterales cases, Osaka, Japan, 2013–2014 and eight other isolates in the database. Plasmid clustering was performed on the basis of gene presence–absence pattern and *intI1* structure (intact or pseudogene). Red and black bars in the heatmap indicate gene presence and absence, respectively. Information on the samples is listed in the table to the right of the heatmap. Information on the isolates from the database that had no relationship with this outbreak is shown as N/A in the column named ‘ID’. N/A: not available; ST: sequence type by multilocus sequence typing.

S1-PFGE profiles were diverse among the 22 isolates (Fig S1). All visible plasmid DNA bands were excised and sequenced, and sequences of plasmids carrying *bla*_IMP-6_ were compared between the isolates. *bla*_IMP-6_ was detected in 23 plasmids from 22 isolates. Figure 3 shows the results of cluster analysis of the 23 plasmids in this study and eight completely sequenced IncN plasmids from epidemiologically unrelated isolates from Japan, China and Taiwan^27–30^. The 23 plasmids in this study were clustered into two types, A (n = 13) and E (n = 6), and four other types with a single plasmid in each (B, C, D and F). All plasmids excluding type F contained a highly conserved IncN plasmid backbone of approximately 50 kb in length. Type E plasmids consisted mainly of an IncN and IncR plasmid backbone containing unique regions and were approximately 101 kb to 138 kb in length. Moreover, type A and E were classified to subtypes A1 (n = 8) and A2 (n = 5) and E1 (n = 4) and E2 (n = 2), respectively, by *ΔintI1* (I*S26* element insertion) and intact *intI1* on the conserved IncN plasmid backbone region. Although three plasmids (types B, C and D) shared an IncN plasmid region, unique structures were detected in each plasmid. One plasmid belonging to type F consisted of an IncFIB plasmid. However, the comparative plasmid analysis indicated that gene composition on IncN plasmids was well conserved among 30 plasmids isolated from China, Taiwan and Japan. The composition of AMR genes and conjugation-related genes and AMR genes, however, showed different patterns among these geographic locations (Fig. S2A,B). Especially, *bla*_CTX-M-2_ was not detected in seven plasmids isolated from China or Taiwan, and four plasmids (pIMP-SZ1501, pIMP-GZ1517, pIMP-SH1506 and pIMP-HK1500) isolated from China possessed *bla*_IMP-4_ instead of *bla*_IMP-6_ (Figs. S2B and 3). Although pKPI-6 (AB616660), which was isolated from Japan in 2008, belonged in type A, pKPI-6 indicated a deletion of 5177 bp in the highly conserved IncN backbone in this study. Furthermore, the conjugation experiments *in vitro* were performed using the representative isolates harbouring type A1 or A2 plasmid as the donor (strain MRY14-211, MRY14-225, MRY14-168 and MRY14-226). Transconjugants with *bla*_IMP-6_ were successfully obtained.

### Epidemiological link by plasmid type

Cases with type A1 plasmid: Cases 1, 2, 3, 5 and 7 were from the Department of Surgery and had an epidemiological link with hospital ward E9 and W9 (Fig. 1 and cases denoted by superscript ‘a’ in Fig. 3), and their isolates were *E. coli* ST357, *E. coli* ST38, *E. coli* ST131 with different PFGE profiles, *E. cloacae* and *K. oxytoca*, respectively. These five isolates shared type A1 plasmid, which was consistent with the epidemiological data.

Cases with type A2 plasmid: Cases 9, 12 and 13 were from the Department of Neurosurgery and had an epidemiological link with ward E11 (Fig. 1 and denoted by superscript ‘b’ in Fig. 3). Although the bacterial species of IMP-6-CPE isolated from these cases were different, *K. aerogenes*, *E. cloacae* and *K. pneumoniae*, these isolates shared type A2 plasmid.

Cases with type E plasmids: Among the five cases with the isolates of clonally related *K. oxytoca* (Cases 5, 17, 18, 20, and 21, Fig. 2b), three cases had an epidemiological link with ward E9 and W9 (Cases 5, 17 and 18). These three isolates and the isolate of *K. pneumoniae* from the Department of Surgery (Case 19) shared type E1 plasmid (denoted by superscript ‘c’ in Fig. 3). The other two isolates of *K. oxytoca* from cases with no apparent epidemiological link with those described above had type E2 plasmid (Cases 20 and 21).

### Case-control study

The case-control study showed that pancreatoduodenectomy (PD: adjusted OR [aOR]=9.5, 95% confidence interval [CI] 1.3–118.5), changing drains in the fluoroscopy room (aOR = 14.3, 95% CI 1.9–∞), continuous peritoneal lavage (aOR = 7.9, 95% CI 1.0–113.2) and enteric fistula (aOR = 8.0, 95% CI 1.5–41.9) were associated with IMP-6-CPE acquisition after adjustment by duration of hospitalization (Table 1)^31^.

**Table 1:** Risk factors of acquiring IMP-6 carbapenemase-producing Enterobacterales among cases with abdominal surgery, Osaka, Japan, 2013–2014.

Factors	Case	(%)	Median	Control	(%)	Median	OR	95%CI	p	aOR*	95%CI	p
	n = 11		(IQR)	n = 24		(IQR)						
Age, years old			76 (65–78)			71 (65–77.5)			0.44			0.32
Male gender	9	(82)		15	(63)		2.7	(0.4–30.4)	0.44	2.5	(0.4–14.5)	0.31
ASA score			2 (2–2)			2 (2–2.5)			0.22			0.11
Diabetes mellitus	1	(9)		2	(8)		1.1	(0.0–23.5)	1.00	1.0	(0.1–12.3)	0.99
Endoscopy within the past 6 months	8	(73)		18	(75)		0.9	(0.1–6.9)	1.00	0.8	(0.2–4.2)	0.81
Room share with cases	7	(64)		10	(42)		2.5	(0.5–14.4)	0.29	2.2	(0.5–10.0)	0.30
ICU admission	9	(82)		16	(67)		2.3	(0.3–25.7)	0.45	2.6	(0.4–17.0)	0.32
ICU admission days			2 (1–4)			1 (0–3.5)			0.34			0.33
Pancreato-duodenectomy	6	(55)		4	(17)		6.0	(0.9–40.0)	0.04	6.4	(1.3–32.4)	0.03
Surgical site infection	11	(100)		19	(79)		—	(0.7 −)	0.16	—	—	—
Changing drains at fluoroscopy room	11	(100)		13	(54)		—	(2.2 −)	<0.01	—	—	—
Continuous peritoneal lavage	9	(82)		10	(42)		6.3	(0.9–68.6)	0.04	5.9	(1.0–34.8)	0.05
Arterial line	11	(100)		19	(80)		—	(0.7 −)	0.16	—	—	—
Central venous line	10	(91)		15	(63)		6.0	(0.6–288.8)	0.12	5.4	(0.6–51.1)	0.14
Enteric fistula	7	(64)		5	(21)		6.7	(1.1–43.1)	0.02	8.0	(1.5–41.9)	0.01
Stoma	1	(9)		10	(42)		0.1	(<0.1–1.3)	0.11	0.2	(<0.1–1.4)	0.10
Enteral feeding	6	(55)		8	(33)		2.4	(0.4–13.2)	0.28	2.6	(0.6–11.4)	0.22
Carbapenem use	2	(18)		10	(42)		0.3	(<0.1–2.1)	0.26	0.3	(0.1–1.8)	0.19
Number of cultures			7.0 (4–9)			7.5 (4.5–11.5)			0.37			
Days of hospitalization			20 (6–26)			17 (6–34.5)			0.80			

*Adjusted by days of hospitalization.

^†^The univariate analyses of these variables were conducted by conditional logistic regression.

IQR: interquartile range; CI: confidence interval; QR: odds ratio; aOR: adjusted odds ratio; ASA: American Society of Anesthesiology; ICU: intensive care unit.

### Infection prevention and responses

Suboptimal standard precautions were observed in several procedures in the wards of the Department of Surgery, including changing drains in the fluoroscopy room and continuous peritoneal lavage. Across the hospital, plastic containers to collect waste fluids from nasogastric tubes, surgical drains or urine were shared among patients within wards with suboptimal disinfection. Infection control and prevention were strengthened together, strict indications for continuous peritoneal lavage were introduced, and the sharing of plastic containers for fluid/urine collection was prohibited. OCPHO coordinated the national and regional infection control experts and supported local CRE surveillance activities. No additional cases were identified as of July 2016, nearly two years after the investigation ended.

## Discussion

In the present study, we described a long-standing multispecies outbreak of IMP-6-CPE in an acute care hospital in Japan. We also highlighted possible epidemiological links among patients with IMP-6-CPE through transmission of the plasmid IncN backbone shared with *bla*_IMP-6_ and possible risk factors for IMP-6-CPE acquisition.

Plasmid analysis by WGS revealed the detailed relationship between cases with IMP-6-CPE and its routes of transmission. In the past, cases infected by different species or by isolates having different PFGE profiles were considered to have no relationship even though those cases had epidemiological links. However, through plasmid analysis by WGS, we discovered hidden relationships (type A1 or A2). And for cases with the same species with related PFGE profiles but having different epidemiological links, plasmid analysis was able to distinguish these differences (type E1 and E2), which suggests a different transmission pattern. Because of the long duration of the event, plasmid analysis allowed tracing of only some of the cases in this study, but the discovery of even some of the links was of great help in understanding the features of the outbreak.

The IncN plasmid appears to have played a key role in *bla*_IMP-6_ transmission in this outbreak although we also observed clonal spread of species. We confirmed that *E. coli* DH10B transconjugants with *bla*_IMP-6_ from the isolates harbouring type A1 or A2 plasmid were successfully obtained *in vitro*. Therefore, this finding indicates that the IncN plasmids carrying *bla*_IMP-6_ in this study are conjugative. The IncN plasmid is a conjugative plasmid reported to be part of a broad-host-range group^32,33^. In the current event, the *bla*_IMP-6_ on the IncN plasmid transmitted among five species of Enterobacterales, and similar multispecies outbreak of *bla*_IMP-4_ harboring Gram-negative bacteria occurred in Australia that involved in *Pseudomonas aeruginosa* and four species of Enterobacterales^34^. The *bla*_IMP-6_-positive Japanese indigenous IncN plasmid in the current event was different from the *bla*_IMP-4_-positive IncN epidemic plasmid in China^27^. There might be a geographical difference in the gene cassette in the IncN plasmid. Long duration of the event with frequent transmissions is one reason why the IncN harbouring *bla*_IMP-6_ spread so many different Enterobacteriaceae species in this event. However, several factors may have an influence on limiting the host-range of plasmids such as interactions with and dependence on host-encoded DNA replication proteins, ability of conjugation and existence of functional modules in plasmids^33^. The observation of epidemiology and the relationship between genera, species, sequence types and plasmids types in each outbreak is essential for understanding geographical diversity and the evolution of the host-range of plasmids, and will help to achieve better control of CPE.

The case-control study showed that PD, changing drains in the fluoroscopy room, continuous peritoneal lavage and enteric fistulae were associated with acquisition of IMP-6-CPE that carried *bla*_IMP-6_. The strength of this case-control study is that the association we evaluated was based on genotype, not phenotype. Therefore, the association we evaluated was the risk of *bla*_IMP-6_ transmission. We adjusted time at risk because it affects the acquisition of multi-drug resistant organisms^31^, but not comorbidities, because the American Society of Anesthesiologists scores of the cases and controls were similar. It is reasonable that PD, one of the most invasive surgeries in which many medical devices are used, was a risk factor because surgery and the use of invasive medical devices were reported to be risk factors of CRE acquisition^35,36^. Poor hand hygiene of healthcare workers observed during the investigation may explain the high ORs for changing drains in the fluoroscopy room and managing the enteric fistula, two medical procedures that frequently accompany PD. From our observation, continuous peritoneal lavage also had the potential to transmit IMP-6-CPE. This is a medical procedure used for severe acute pancreatitis or necrotizing pancreatitis^37,38^, but its effectiveness and safety have not been studied sufficiently. It requires a complex water-handling system in which the maintenance of sterile conditions is difficult and thus might also have an influence on IMP-6-CPE transmission. Further studies are needed to evaluate the effectiveness and safety of this procedure, and to consider its risk-benefit. Several reports have shown that endoscopy can transmit CRE^39^, but endoscopy use within the past 6 months was not associated with IMP-6-CPE acquisition in this study.

OCPHO played a pivotal role in coordinating hospital, laboratories, regional and national rapid response teams and in the implementation of local CRE surveillance. One study reported that 12.2% of patients screened were positive for CRE in Osaka after the outbreak in ONH^40^. This finding suggests that CRE might have already been prevalent in the area. The unique characteristics of IMP-6-CPE showing susceptibility to imipenem would also require antimicrobial susceptibility testing for meropenem in this area. The importance of coordinated, sustainable surveillance supported by local public health centres was also stressed in many countries in the regions of the Americas, Europe, and Asia^41–46^. The outbreak in ONH also affected the national infectious diseases surveillance system as CRE infection became one of the notifiable diseases under Infectious Diseases Control Law in Japan from September 2014^47^. In this notification, carbapenem resistance was defined as a MIC of meropenem ≧2 μg/mL or imipenem ≧2 μg/mL plus that of cefmetazole ≧64 μg/mL. In 2016, 1573 cases of CRE infection were notified^48^, and we started to feedback the data on the website of the institution to ensure a timely response to the event by hospitals and local public health centres.

There are several limitations to the interpretation of the present results. First, the discovery of cases was dependent on the clinical culture and sampling policy, which varied across the hospital, and some cases might have been missed. However, samples were frequently obtained and cultured in the Department of Surgery. Additionally, active screening for those patients with invasive devices was conducted in March 2014, and the situation was comprehensively evaluated at that point. Second, suspected MBL producer positive cases in which IMP-6 was not detected were excluded from the study. The screening for MBL tested positive at ONH, and the resistance gene could have been lost during subculturing and preservation. Third, the controls in the case-control study were selected based on their phenotype, and the absence of *bla*_IMP-6_ was not confirmed. However, this selection process can result in non-differential misclassification and could bias towards null association.

In this study, we described possible epidemiological links and risk factors for patients with IMP-6-CPE acquired in a single hospital by analysis of the plasmid backbone shared with *bla*_IMP-6_. The outbreak terminated through coordinated responses with the hospital and local public health centre, even after transmission in the hospital for years. These findings provide clues to controlling CPE outbreaks in healthcare settings.

## Supplementary information

Supplementary information.
