# Supplementary material for: A prolonged multispecies outbreak of IMP-6 carbapenemase-producing Enterobacterales due to horizontal transmission of the IncN plasmid
Source: Sci Rep. 2020 Mar 5;10:4139. doi: 10.1038/s41598-020-60659-2 (PMC7057946; doi:10.1038/s41598-020-60659-2)
Supplement: Supplementary file 1 — Supplementary information. [file 41598_2020_60659_MOESM1_ESM.pdf]

Title:

A prolonged multispecies outbreak of IMP-6 carbapenemase-producing Enterobacterales due to horizontal transmission of the IncN plasmid

Authors: Takuya Yamagishi, Mari Matsui, Tsuyoshi Sekizuka, Hiroaki Ito, Munehisa Fukusumi, Tomoko Uehira, Miyuki Tsubokura, Yoshihiko Ogawa,

Atsushi Miyamoto, Shoji Nakamori, Akio Tawa, Takahisa Yoshimura, Hideki Yoshida, Hidetetsu Hirokawa,

Satowa Suzuki, Tamano Matsui, Keigo Shibayama, Makoto Kuroda & Kazunori Oishi

Figure S1. Pulsed-field gel electrophoresis profiles of S1 nuclease-treated DNA of the 22 isolates

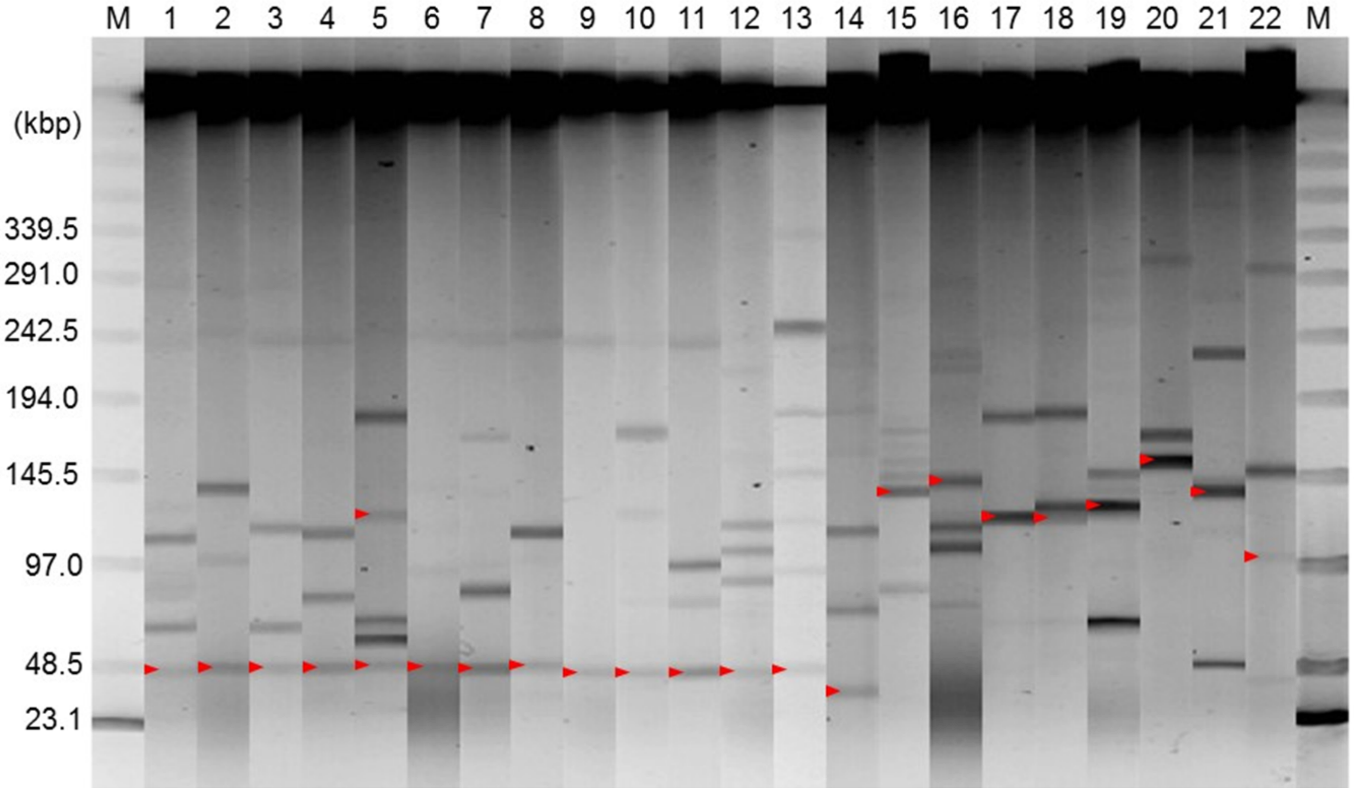

The lanes were normalized and rearranged by GelComper II software, version 6.6 (Applied Maths, St-Martens-Latem, Belgium). Bands of plasmid carrying *bla*$_{IMP-6}$ are indicated by red arrowheads. Lane M: CHEF DNA size standards lambda ladder (Bio-Rad, Hercules, CA, USA) and □-Hind III digest (TaKaRa Bio, Shiga, Japan). Lanes 1-22: isolates from cases 1 to 22 in Figure 1, respectively.

Figure S2. Comparative analysis of IncN plasmid region and AMR gene patterns.

A)

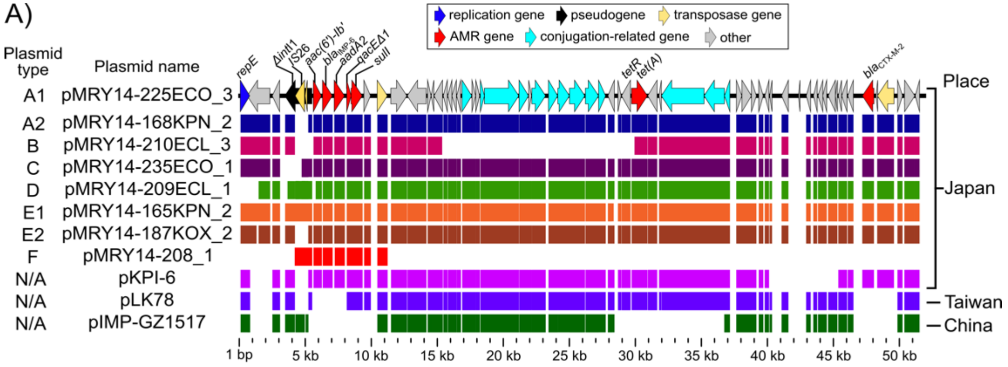

B)

Plasmid type	Species	Plasmid replicon type	*IS26* in *intI1*	Place	Collection date	Plasmid name	Accession number	*aac(6')-Ib'*	*bla*IMP-6	*aadA2*	*qacEΔ1*	*sul1*	*tet(A)*	*bla*CTX-M-2	*bla*TEM-1	*aac(6')-Ib-cr*	*aadA16*	*arr-3*	*bla*NDM-1	*bla*OXA-10	*ble*	*dfrA27*	*bla*CTX-M-24	*fosA5*	*qnrA1*	*bla*IMP-4	*qnrS1*	*dfrA1*	*bla*LAP-2	*aac(3)-IIa*	*bla*CTX-M-15	*bla*OXA-1
								AMR gene																								
A1	*E. coli*	N	+	Japan	Oct 2013	pMRY14-225ECO_3	AP018745	+	+	+	+	+	+	+	-	-	-	-	-	-	-	-	-	-	-	-	-	-	-	-	-	-
	E. cloacae	N	+	Japan	Nov 2013	pMRY14-211ECL_2	AP018744	+	+	+	+	+	+	+	-	-	-	-	-	-	-	-	-	-	-	-	-	-	-	-	-	-
	E. coli	N	+	Japan	Dec 2013	pMRY14-229ECO_3	AP018746	+	+	+	+	+	+	+	-	-	-	-	-	-	-	-	-	-	-	-	-	-	-	-	-	-
	E. coli	N	+	Japan	Dec 2013	pMRY14-230ECO_3	AP019252-253	+	+	+	+	+	+	+	-	-	-	-	-	-	-	-	-	-	-	-	-	-	-	-	-	-
	K. oxytoca	N	+	Japan	Dec 2013	pMRY14-192KOX_5	AP019230-234	+	+	+	+	+	+	+	-	-	-	-	-	-	-	-	-	-	-	-	-	-	-	-	-	-
	E. coli	N	+	Japan	Feb 2014	pMRY14-457ECO_1	AP019291-294	+	+	+	+	+	+	+	-	-	-	-	-	-	-	-	-	-	-	-	-	-	-	-	-	-
	E. coli	N	+	Japan	Dec 2013	pMRY14-231ECO_2	AP019254-257	+	+	+	+	+	+	+	-	-	-	-	-	-	-	-	-	-	-	-	-	-	-	-	-	-
	E. coli	N	+	Japan	Nov 2013	pMRY14-228ECO_2	AP019249-251	+	+	+	+	+	+	+	-	-	-	-	-	-	-	-	-	-	-	-	-	-	-	-	-	-
A2	*K. aerogenes*	N	-	Japan	Mar 2014	pMRY14-243KAE_1	AP019272-273	+	+	+	+	+	+	+	-	-	-	-	-	-	-	-	-	-	-	-	-	-	-	-	-	-
	K. pneumoniae	N	-	Japan	Dec 2013	pMRY14-168KPN_2	AP018742	+	+	+	+	+	+	+	-	-	-	-	-	-	-	-	-	-	-	-	-	-	-	-	-	-
	E. coli	N	-	Japan	Oct 2013	pMRY14-226ECO_3	AP019247-248	+	+	+	+	+	+	+	-	-	-	-	-	-	-	-	-	-	-	-	-	-	-	-	-	-
	E. cloacae	N	-	Japan	Feb 2014	pMRY14-233ECL_4	AP019258-260	+	+	+	+	+	+	+	-	-	-	-	-	-	-	-	-	-	-	-	-	-	-	-	-	-
	K. pneumoniae	N	-	Japan	Nov 2013	pMRY15-537KPN_2	AP019295	+	+	+	+	+	+	+	-	-	-	-	-	-	-	-	-	-	-	-	-	-	-	-	-	-
N/A	*K. pneumoniae*	N	-	Japan	2008	pKPI-6	AB616660	+	+	+	+	+	+	+	-	-	-	-	-	-	-	-	-	-	-	-	-	-	-	-	-	-
B	*E. cloacae*	N	-	Japan	Oct 2013	pMRY14-210ECL_3	AP018743	+	+	+	+	+	p	+	-	-	-	-	-	-	-	-	-	-	-	-	-	-	-	-	-	-
N/A	*E. coli*	N	-	Taiwan	Sep 2012	pLK75	KJ440076	-	-	-	+	+	+	-	-	+	+	+	+	+	+	+	-	-	-	-	-	-	-	-	-	-
	K. pneumoniae	N	-	Taiwan	Sep 2012	pLK78	KJ440075	-	-	-	+	+	+	-	-	+	+	+	+	+	+	+	-	-	-	-	-	-	-	-	-	-
	K. pneumoniae	N	-	China	2002	pKP96	EU195449	-	-	-	+	+	+	-	-	+	-	-	-	-	-	-	+	+	+	-	-	-	-	-	-	-
C	*E. coli*	N, FIA, FII	+	Japan	Jan 2014	pMRY14-235ECO_1	AP019261-271	+	+	+	+	+	+	+	+	-	-	-	-	-	-	-	-	-	-	-	-	-	-	-	-	-
D	*E. cloacae*	untype	+	Japan	Sep 2013	pMRY14-209ECL_1	AP019241-246	p	+	+	+	+	+	+	-	-	-	-	-	-	-	-	-	-	-	-	-	-	-	-	-	-
N/A	*K. pneumoniae*	N	+	China	Jan 2012	pIMP-SZ1501	KU051708	-	-	-	-	-	-	-	-	-	-	-	-	-	-	-	-	-	-	+	+	-	-	-	-	-
	E. coli	N	+	China	Sep 2013	pIMP-GZ1517	KT982618	-	-	-	-	-	-	-	-	-	-	-	-	-	-	-	-	-	-	+	+	-	-	-	-	-
	E. cloacae	N	+	China	Aug 2012	pIMP-SH1506	KT989598	-	-	-	-	-	-	-	-	-	-	-	-	-	-	-	-	-	-	+	+	-	-	-	-	-
	C. freundii	N	+	China	Nov 2011	pIMP-HK1500	KT989599	-	-	-	-	-	-	-	-	-	-	-	-	-	-	-	-	-	-	+	+	-	-	-	-	-
E1	*K. oxytoca*	N, R	+	Japan	Jan 2014	pMRY14-193KOX_2	AP019235-238	+	+	+	+	+	+	+	-	-	-	-	-	-	-	-	-	-	-	-	+	+	+	-	-	-
	K. oxytoca	N, R	+	Japan	Nov 2013	pMRY14-191KOX_2	AP019210-215	+	+	+	+	+	+	+	-	-	-	-	-	-	-	-	-	-	-	-	+	+	+	-	-	-
	K. pneumoniae	N, R	+	Japan	Jul 2013	pMRY14-165KPN_2	AP018741	+	+	+	+	+	+	+	-	-	-	-	-	-	-	-	-	-	-	-	-	+	+	-	-	-
	K. oxytoca	N, R	+	Japan	Dec 2013	pMRY14-192KOX_2	AP019216-229	+	+	+	+	+	+	+	-	-	-	-	-	-	-	-	-	-	-	-	+	+	+	-	-	-
E2	*K. oxytoca*	N, R	-	Japan	Jul 2013	pMRY14-187KOX_2	AP019199-209	+	+	+	+	+	+	+	-	-	-	-	-	-	-	-	-	-	-	-	+	+	+	-	-	-
	K. oxytoca	N, R	-	Japan	Mar 2014	pMRY14-247KOX_2	AP019274-290	+	+	+	+	+	+	+	-	-	-	-	-	-	-	-	-	-	-	-	+	+	+	+	+	+
F	*E. cloacae*	FIB	+	Japan	Sep 2013	pMRY14-208ECL_1	AP019239-240	+	+	+	+	+	-	-	-	-	-	-	-	-	-	-	-	-	-	-	-	-	-	-	-	-

+, positive; -, negative; p, pseudogene.

A) Comparative analysis of IncN plasmid region using BLAST atlas. The coding regions of pMRY14-225ECO_3 were aligned against those of 10 other plasmids using TBLASTX, followed by visualisation using GView server. The coloured bars show the complete identity of translated amino acid sequences between ORF regions in the reference plasmid and the other plasmid sequences. Key: N/A, not available. B) Comparison of AMR gene patterns among 31 plasmids. The AMR genes of plasmids were analysed by ResFinder program. Key: N/A, not available; +, positive; -, negative; p, pseudogene.

Supplementary Table. Plasmid sequence information in this study

Organism	Strain name	Plasmid name	PBRT Inc type	Sequence status	Number of contigs	Total contig size (bp)	S1-PFGE band size (kb)	Ratio (contig size/band size)	Accession number	ID
E. cloacae	MRY14-211	pMRY14-211ECL_2	N	Complete	1	51,983	40	1.30	AP018744	2
E. cloacae	MRY14-210	pMRY14-210ECL_3	N	Complete	1	40,793	30	1.36	AP018743	14
E. cloacae	MRY14-233	pMRY14-233ECL_4	N	Draft	3	56,528	44	1.28	AP019258-AP019260	12
E. cloacae	MRY14-209	pMRY14-209ECL_1	Untyped	Draft	6	144,027	150	0.96	AP019241-AP019246	16
E. cloacae	MRY14-208	pMRY14-208ECL_1	FIB	Draft	2	104,686	100	1.05	AP019239-AP019240	22
E. coli	MRY14-229	pMRY14-229ECO_3	N	Complete	1	51,998	45	1.16	AP018746	3
E. coli	MRY14-225	pMRY14-225ECO_3	N	Complete	1	51,983	50	1.04	AP018745	1
E. coli	MRY14-228	pMRY14-228ECO_2	N	Draft	3	57,776	45	1.28	AP019249-AP019251	8
E. coli	MRY14-231	pMRY14-231ECO_2	N	Draft	4	55,725	45	1.24	AP019254-AP019257	7
E. coli	MRY14-230	pMRY14-230ECO_3	N	Draft	2	51,761	45	1.15	AP019252-AP019253	4
E. coli	MRY14-457	pMRY14-457ECO_1	N	Draft	4	50,875	40	1.27	AP019291-AP019294	6
E. coli	MRY14-226	pMRY14-226ECO_3	N	Draft	2	50,590	45	1.12	AP019247-AP019248	11
E. coli	MRY14-235	pMRY14-235ECO_1	N, FIA, FII	Draft	11	139,357	140	1.00	AP019261-AP019271	15
K. aerogenes	MRY14-243	pMRY14-243KAE_1	N	Draft	2	51,158	50	1.02	AP019272-AP019273	9
K. oxytoca	MRY14-192	pMRY14-192KOX_5	N	Draft	5	53,712	40	1.34	AP019230-AP019234	5.1
K. oxytoca	MRY14-192	pMRY14-192KOX_2	N, R	Draft	14	129,584	130	1.00	AP019216-AP019229	5.2
K. oxytoca	MRY14-187	pMRY14-187KOX_2	N, R	Draft	11	138,178	160	0.86	AP019199-AP019209	20
K. oxytoca	MRY14-247	pMRY14-247KOX_2	N, R	Draft	17	119,351	140	0.85	AP019274-AP019290	21
K. oxytoca	MRY14-193	pMRY14-193KOX_2	N, R	Draft	4	103,391	130	0.80	AP019235-AP019238	17
K. oxytoca	MRY14-191	pMRY14-191KOX_2	N, R	Draft	6	100,909	100	1.01	AP019210-AP019215	18
K. pneumoniae	MRY14-168	pMRY14-168KPN_2	N	Complete	1	50,951	40	1.27	AP018742	10
K. pneumoniae	MRY15-537	pMRY15-537KPN_2	N	Draft	1	52,178	46	1.13	AP019295	13
K. pneumoniae	MRY14-165	pMRY14-165KPN_2	N, R	Complete	1	130,480	130	1.00	AP018741	19

PBRT: PCR-based replicon typing; PFGE: pulsed field gel electrophoresis.
